# All creatures great and small: celebrating the microbiome

**DOI:** 10.1038/s42003-021-02299-4

**Published:** 2021-06-25

## Abstract

The collection of bacteria, fungi, and other microbes that form microbiota play an important role in both human and environmental health. In recognition of World Microbiome Day, we have curated a Collection of articles, news and commentary to celebrate the diversity of microbiome research published at *Communications Biology*, and highlight exciting new avenues for the field.

Microbiome research can be traced back to the late 17th century when, armed with a home-made microscope, Antonie van Leeuwenhoek presented the first known descriptions of the human microbiota^1^. While van Leeuwenhoek’s letter only described a handful of microbes (or as he called them, animalcules^1^), the development of next-generation sequencing has enabled modern microbiologists to examine thousands of microbial taxa in a single sample.

“Since its launch in January 2018, *Communications Biology* has aimed to provide a home for microbiome research in its many forms.”

These advances have empowered scientists to explore the microbiome in unprecedented detail, starting with microbial taxa that colonize the human gut. We are grateful to have published many exciting gut microbiome papers that examine how intestinal taxa respond to diet, promote normal health, or even predict disease. In fact, our most-cited microbiome paper to date was from Simone Rampelli et al.^2^, who reported that changes in the gut microbiome of children could potentially serve as a biomarker for obesity. Coincidentally, our most-accessed microbiome paper also came from Simone Rampelli et al.^3^, who reconstructed a Neanderthal gut microbiome from paleofecal samples in El Salt, Spain. Looking beyond just bacteria, some of our papers have also highlighted the role of the gut mycobiome in mouse diet and metabolism^4^, or gut mycobiome dysbiosis in patients infected with COVID-19 or H1N1^5^. One exciting extension of this line of microbiome research is the gut-brain axis, or how microbes can impact host neurobiology. While gut-brain studies have largely been limited to animal models, we are particularly excited to review potential insights into how bacterial taxa can contribute to neurological disease or behavior.

Of course, the microbiome is not exclusive to humans, and we are fortunate to have published studies on how microbiota impact invertebrate species like coral^6^, bees^7^, or termites^8^. We also recognize that environmental microbiota play an important role in maintaining ecosystems ranging from the Brazilian Cerrado^9^ to the Great Barrier Reef^10^. Keeping in mind that this year’s theme for World Microbiome Day is sustainability, we believe it is especially important to understand how natural microbiota might be leveraged to promote environmental health. Some of these discoveries may come from unlikely places, as covered in a recent Research Highlight^11,12^ on how methanotrophic taxa in bark may limit methane emissions from trees. Yuya Sato et al.^13^ also reported that ammonia- and nitrate-oxidizing bacteria may play a key role in degrading oils at wastewater treatment plants, hinting at potential taxa for improved sustainability efforts.

Since its launch in January 2018, *Communications Biology* has aimed to provide a home for microbiome research in its many forms. Our Collection published today illustrates the breadth of our current microbiome research, and we remain excited to follow the advances in this field, including studies that explore the role of microbiota in the gut-brain axis or environmental health.

**Figure:**
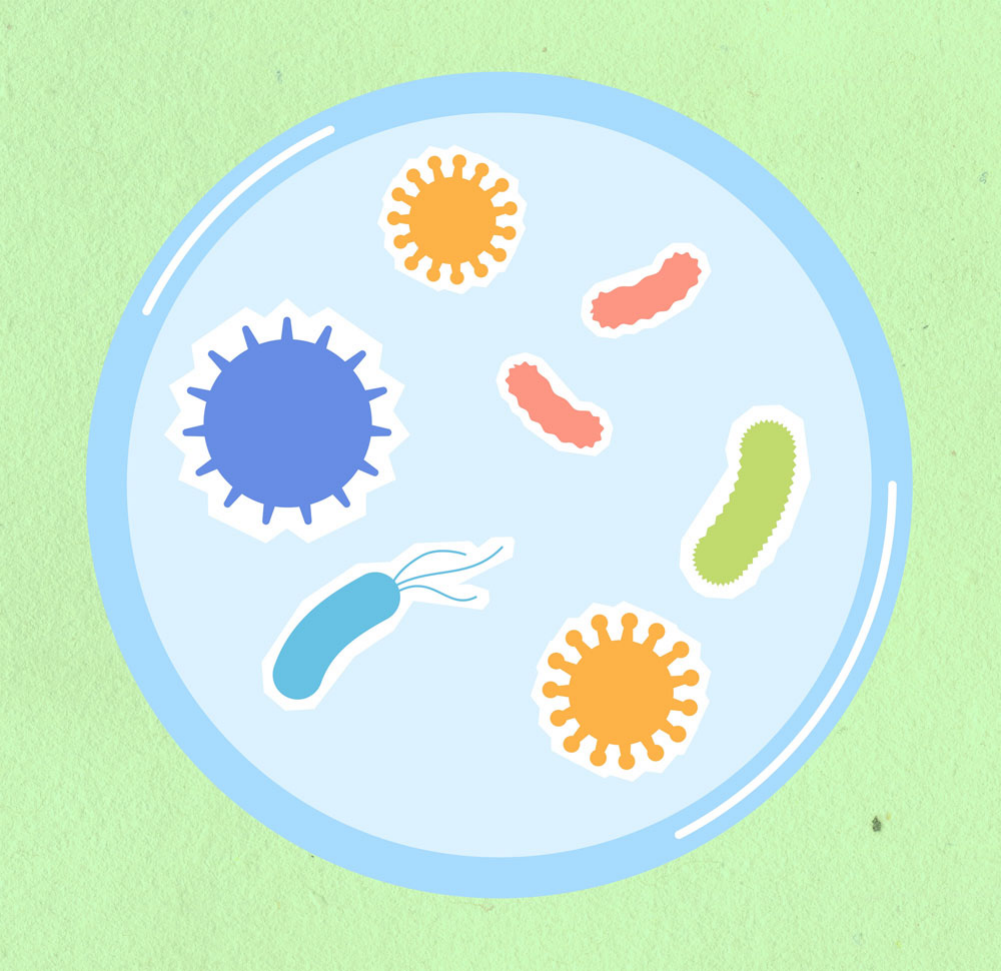
Pexels
